# Hyperosmotic stress induces PARP1‐mediated HPF1‐dependent mono(ADP‐ribosyl)ation

**DOI:** 10.1002/1873-3468.70334

**Published:** 2026-03-31

**Authors:** Anna Georgina Kopasz, Mihály Mérey, Rebeka Vásárhelyi, Ramóna Pék, Victor Imburchia, László Henn, Adrián Kószó, Nicholas D. Lakin, Ivan Ahel, Sébastien Huet, Ágnes Czibula, Gyula Timinszky

**Affiliations:** ^1^ Laboratory of DNA Damage and Nuclear Dynamics, Institute of Genetics, HUN‐REN Biological Research Centre Szeged Hungary; ^2^ Doctoral School of Multidisciplinary Medical Sciences University of Szeged Hungary; ^3^ Doctoral School of Biology, Faculty of Science and Informatics University of Szeged Hungary; ^4^ Univ Rennes, CNRS, IGDR (Institut de Génétique et Développement de Rennes) – UMR 6290, BIOSIT (Biologie, Santé, Innovation Technologique de Rennes) – UMS 3480 France; ^5^ Department of Biochemistry University of Oxford UK; ^6^ Sir William Dunn School of Pathology University of Oxford UK; ^7^ Department of Immunology, Albert Szent‐Györgyi Medical School University of Szeged Hungary

**Keywords:** HPF1, hyperosmotic stress, mono(ADP‐ribosyl)ation, PARP1

## Abstract

While the downstream effectors of the hyperosmotic stress response are relatively well characterized, the primary molecular sensors responsible for initial stress detection remain poorly defined. In this study, we demonstrate that hyperosmotic stress triggers a rapid and transient mono(ADP‐ribosyl)ation (MARylation). Beside MARylation, signs of acute genotoxicity are missing and CHK1 activation is observed only upon recovery from osmotic stress. Our data indicate that PARP1 catalyzes its own MARylation in an HPF1 co‐factor dependent manner. Biochemical assays further demonstrate that the mono‐ADP‐ribose moiety is resistant to hydroxylamine treatment, which is a feature of HPF1‐directed O‐glycosidic bonds. Together, these findings support a model in which PARP1 acts as a sensor of chromatin structure changes induced by hyperosmotic stress leading to its autoMARylation.

## Graphical abstract

Sorbitol‐induced hyperosmotic stress rapidly induces reversible mono(ADP‐ribosyl)ation (MARylation) on PARP1 without the signs of genotoxic signaling. We show that PARP1 autoMARylation is HPF1 dependent and forms hydroxylamine‐resistant O‐glycosidic linkages. Our model suggests that PARP1 senses chromatin structural changes induced by hyperosmotic stress without signs of acute genotoxicity. Created in biorender. Kopasz, A. (2026) https://BioRender.com/n4amwze.

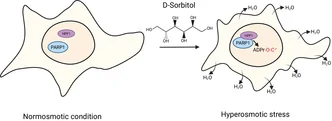

## Abbreviations

**ADPr**, ADP‐ribose

**ARH3**, (ADP‐ribosyl)hydrolase 3

**ASK3**, apoptosis signal‐regulating kinase 3

**ATR**, Ataxia‐Telangiectasia and RAD3‐Related Serine/Threonine Kinase

**CHK1**, Checkpoint Kinase 1

**CPT**, camptothecin

**DSBs**, double‐stranded breaks

**G4 DNA**, G‐quadruplex secondary DNA structures

**HA**, hydroxylamine

**HPF1**, Histone PARylation Factor 1

**γH2AX**, Phosphorylation of histone H2AX

**GAPDH**, Glyceraldehyde‐3‐Phosphate Dehydrogenase

**MEF**, mouse embryonic fibroblast

**MAR**, mono(ADP‐ribose)

**MRE11**, meiotic recombination 11

**PARG**, poly(ADP‐ribose) glycohydrolase

**PARP**, poly(ADP‐ribose) polymerase

**ROS**, reactive oxygen species

**SNP**, single‐nucleotide polymorphism

**SSBs**, single‐stranded breaks

**U2OS**, Uppsala Osteosarcoma cell line

Hyperosmotic stress is defined as the increased osmolality of the extracellular fluid compared to the intracellular one affecting both unicellular organisms, as well as various cell types of multicellular organisms [1, 2]. While the effects of broad osmotic changes on renal cells of the inner medullary region of the kidney are the best characterized [3, 4], there is growing evidence of its relevance in multiple organ systems [5, 6]. In mammals, physiological osmolality is fine‐tuned in a range of 285 to 295 mOsm·kg^−1^ of H_2_O via maintaining serum homeostasis [7], and increased osmolality leads to diverse acute and chronic illnesses, primarily through the release of pro‐inflammatory cytokines [8, 9].

Hyperosmosis immediately leads to decreased cell volume and increased concentrations of intracellular components and changes chromatin organization [10, 11]. These changes act through mechanisms such as macromolecular crowding and cytoskeletal perturbation, profoundly affecting biochemical processes and contributing to increased reactive oxygen species (ROS) generation and DNA damage [12, 13]. Cellular adaptation strategies to hyperosmotic stress and the response at the level of downstream effectors are broadly studied; however, the initial sensors recognizing different cellular stressors remain poorly understood.

PARP1 is a first‐line responder of oxidative stress and DNA damage. By using NAD^+^ as a substrate, PARP1 attaches either a single ADP‐ribose (ADPr) moiety to the target amino acids called mono(ADP‐ribosyl)ation or MARylation, or it forms long ADP‐ribose chains composed of hundreds of ADP‐ribose residues called poly(ADP‐ribosyl)ation or PARylation [14]. On its target proteins including itself, PARP1 is able to form acyl‐O‐ester linkage on glutamate and aspartate residues, while in the presence of its co‐factor HPF1, it switches target preference and creates O‐glycosidic bonds with serine and tyrosine amino acids [15, 16, 17].

The biological importance of both PARP1‐mediated mono‐ and poly(ADP‐ribosyl)ation has been described in various studies. PARP1 produces long, branched, negatively charged PAR chains on itself and on histones; these chains serve as a platform that attract PAR‐binding repair proteins and facilitate the repair of single‐stranded breaks (SSBs) and double‐stranded breaks (DSBs) [18, 19, 20, 21, 22, 23, 24, 25]. PARP1‐mediated PARylation also contributes to chromatin remodeling and gives access to the damaged DNA as well as corresponds to regulation of transcriptional responses helping cells adapt to various physiological and pathological conditions [26]. Histone mono‐ADP‐ribosylation has been characterized as a second wave of PARP1 signaling and via the recruitment of RNF114 ubiquitin‐ligase it is able to reprogram the DNA damage site‐signaling [27, 28]. Moreover, PARP1‐mediated PARylation is trimmed by poly(ADP‐ribose) glycohydrolase (PARG) to a single, terminal MAR [29], which is subsequently erased by ARH3 [30] or the macrodomain‐containing mono(ADP‐ribosyl)hydrolases [31, 32]. Notably, recent studies have shown that PARG can remove the terminal MAR from glutamate, aspartate, and tyrosine residues, as well [17, 33, 34].

Beyond protein targets of PARP1, it was shown that PARP1 is able to modify ssDNA and dsDNA ends directly, suggesting a novel role of PARP1 in maintaining genome integrity [35, 36]. In line with biochemical experiments, PARP1 was proved to modify telomere DNA and telomere DNA‐ADPr is enhanced during exposure to genotoxic agents [37].

Apart from safeguarding the genome, PARP1‐driven modifications contribute to the cellular defense mechanisms against genotoxic stress by controlling stress granule assembly in response to hydrogen peroxide, arsenite exposure, or DNA alkylation damage [38, 39, 40]. It was recently shown that the basal PAR level is required to maintain liquid‐phase condensates of ASK3 transducing osmosensing signal into ASK3 inactivation [41]. Inactivation of ASK3 is important to orchestrate cell volume recovery under hyperosmotic stress [42]. However, the role of PARP1 activation and the appearance of *de novo* ADP‐ribosylation during hyperosmotic stress is largely unknown, which inspired us to explore this post‐translational modification in osmotic pressure sensing. Here, we demonstrate that hyperosmotic stress induces rapid, persistent MARylation and short‐lived PARylation that can be reversed by the restoration of normosmotic extracellular conditions. Moreover, we showed that hyperosmotic stress–induced MARylation is PARP1‐driven and HPF1‐dependent.

## Materials and methods

### Cell culture and sample collection

Human U2OS osteosarcoma cells (RRID:CVCL_0042; ATCC HTB‐96), and derivatives were authenticated by STR profiling (Eurofins Genomics, Ebersberg, Germany). U2OS ARH3 KO, HPF1 KO, PARP1 KO, PARP2 KO, and corresponding wild‐type cells were described earlier [43, 44, 45]. To generate U2OS PARP1 KO cells stably expressing mEGFP‐PARP1, cells were transfected with the pmEGFP‐C1‐PARP1 mammalian expression vector [46] and then selected using media supplemented with 500 μg·mL^−1^ G418. Cell lines were cultured in high‐glucose DMEM with L‐glutamine and sodium pyruvate (Capricorn Scientific GmbH, Ebsdorfergrund, Germany) supplemented with 10% FBS (Biosera, Cholet, France) and 1% penicillin–streptomycin (Biosera), in the presence of 5% CO_2_ at 37 °C. Cells were treated with 0.4 m D‐sorbitol (S1876; Sigma‐Aldrich, Saint Louis, MO, USA), 2 mm H_2_O_2_ (Thermo Scientific, Waltham, MA, USA), 5 μm Olaparib (AZD2281; Selleck Chemicals, Houston, TX, USA), 5 nm Saruparib (HY‐132167; MedChem Express, Monmouth Junction, NJ, USA), or 10 μm PARG inhibitor (HY‐108360; MedChem Express) dissolved in complete DMEM. Prior to sample collection, cells were washed with cold 1× PBS, then lysed in 4% SDS lysis buffer (4% SDS, 50 mm Tris–HCl, pH 7.4, 100 mm NaCl, 4 mm MgCl_2_, 5 U·μL^−1^ Benzonase [Pierce™ Universal Nuclease for Cell Lysis, 88700; Thermo Scientific)]. Protein concentration was equalized based on the measurement of the initial concentration by NanoDrop.

### Cell survival assay

U2OS PARP1/2 knockout and wild‐type cells were seeded into 96‐well plates with complete medium (DMEM 10% FBS) and allowed to adhere for 24 h. Subsequently, hyperosmotic stress was induced by treating the cells with 0.4 m D‐sorbitol for 10, 30, or 60 min. Following the treatment, sorbitol‐containing medium was replaced with complete medium, and the cells were incubated for 1 week at 37 °C in a CO_2_ incubator. The culture medium was refreshed on the third day of incubation. Cell viability was assessed using a resazurin assay. One hundred microliters of resazurin solution (25 μg·mL^−1^ in Leibovitz's medium) was added to each well, and the plates were incubated at 37 °C for 30–50 min, until the development of a fluorescent resorufin signal. Fluorescence was measured using a Synergy H1 microplate reader (BioTek Instruments, Winooski, VT, USA) with excitation/emission filters set to 530/590 nm. Cell survival data were presented relative to nontreated controls. Each experimental setup was replicated in three different wells on each plate, and the experiment was independently repeated three times.

### Bis‐tris SDS/PAGE‐based immunoblotting for ADP‐ribose detection

After mixing the samples with NuPAGE 4× LDS Sample Buffer (Invitrogen, Carlsbad, CA, USA), they were resolved on NuPAGE 4 to 12% Bis‐Tris gels (Invitrogen) and ran in NuPAGE 1× MOPS SDS Running Buffer (Invitrogen). Thereafter, proteins were transferred onto a 0.2‐μm nitrocellulose membrane in 1× mPAGE Transfer Buffer (Millipore, Schwalbach, Germany) supplemented with 10% methanol at 4 °C. Alternatively, 8% Bis‐Tris gels (Merck, Darmstadt, Germany) were used along with mPAGE MOPS SDS Running Buffer and mPAGE Transfer Buffer (Merck) supplemented with 10% ethanol. Ponceau S staining was used as a loading control. Membranes were blocked in 5% skim milk in 1× PBS buffer with 0.1% Tween 20 (PBST) for 1 h at room temperature and incubated overnight at 4 °C or 2 h at room temperature in peroxidase‐conjugated mono(ADP‐ribose)‐binding reagents or rabbit poly(ADP‐ribose)‐binding reagent, followed by 1 or 2 h incubation with peroxidase‐conjugated goat anti‐rabbit antibody (Table 1). Immunocomplexes were visualized with the help of ECL Select Western Blotting Detection Reagent (Amersham, Sigma Aldrich, Saint Louis, MO, USA) or Western Bright ECL Spray (Advansta Corp., Menlo Park, CA, USA) in UVITEC Alliance Q9 system or GE Amersham Imager 680.

**Table 1:** Antibodies. Names, origins, vendors, reference numbers (or reference), and dilution of the antibodies used.

Antibodies	Host	Company	References	Dilution
α‐mono‐ADPr (AbD43647)—HRP	‐	Bio‐Rad Lab (Hercules, CA, USA)	TZA020P	1 : 5000
α‐PARP1S499‐ADPr (AbD34251)—HRP	‐	‐	[27]	1.7 μg·mL^−1^
α‐Poly/mono ADPr	Rabbit	Cell Signaling Tech (Danvers, MA, USA)	89 190	1 : 3000
α‐pCHK1 S317	Rabbit	Cell Signaling Tech	2344	1 : 1000
α‐CHK1	Mouse	Cell Signaling Tech	2360	1 : 6000
α‐GAPDH	Rabbit	Proteintech (Rosemont, IL, USA)	10494‐1‐AP	1 : 10 000
α‐γH2AX S139	Mouse	Invitrogen (Carlsbad, CA, USA)	MA‐2022	1 : 2000
α‐PARP1	Rabbit	Abcam (Cambridge, UK)	ab32138	1 : 2000
α‐PARP2	Mouse	Novus Biologicals (Centennial, CO, USA)	NBP2‐89041	1 : 2000
α‐histone H3	Rabbit	Abcam (Cambridge, UK)	ab1791	1 : 5000
α‐Rabbit IgG—HRP	Goat	Invitrogen	G‐21234	1 : 10 000
α‐Mouse IgG—HRP	Goat	Invitrogen	A16066	1 : 3000
α‐Mouse IgG—Alexa 555	Goat	Invitrogen	A21422	1 : 700

### Tris‐glycine SDS/PAGE based immunoblotting

Samples were boiled in NuPAGE 1× LDS Sample Buffer (Invitrogen) for 5 min at 95 °C in the presence of 50 mm DTT. Afterward, samples were loaded onto 12% PAGE (Bio‐Rad Laboratories, Hercules, CA, USA) Tris‐Glycine gels and run in 1× Tris‐Glycine buffer, pH = 8.3 (25 mm Tris base and 192 mm glycine) supplemented with 0.1% SDS. Overnight transfer of proteins was performed onto 0.2 μm nitrocellulose membrane in Tris‐Glycine pH = 8.3 Buffer (20 mm Tris base and 150 mm glycine) supplemented with 20% methanol at 4 °C. Membranes were blocked in 5% skim milk for 60 min, then incubated with rabbit anti‐pCHK1 S317, mouse anti‐CHK1, rabbit anti‐GAPDH, rabbit anti‐PARP1, or mouse anti‐PARP2 primary antibodies overnight at 4 °C. Peroxidase‐conjugated goat anti‐rabbit or goat anti‐mouse secondary incubation was done for 1.5 h at RT on the following day (Table 1). Immunocomplexes were visualized by using Pierce ECL (Thermo Fisher Scientific, Waltham, CA, USA) and detection was done in UVITEC Alliance Q9 system.

### Mono(ADP‐ribose) immunoprecipitation

For the pulldown of MARylated proteins upon hyperosmotic stress we adapted a recently published protocol [47]. Untreated U2OS wide‐type cells or cells exposed to 0.4 m D‐sorbitol for 30 min were collected from two confluent 10 cm dishes by washing twice in 5–5 mL ice‐cold PBS supplemented with 1 μm Olaparib and 1 μm PARG inhibitor (HY‐108360; MedChem Express) then scraping the cells in 250 μL of the same inhibitor‐supplemented PBS. Cell lysis was performed by adding 250 μL of 2× RIPA lysis buffer [100 mm Tris–HCl, pH 7.0; 300 mm NaCl; 2% IP‐40; 1% sodium deoxycholate, 0.2% SDS, 1 μm Olaparib, 1 μm PARG inhibitor, 2 × protease inhibitor cocktail (Protease inhibitor cocktail, EDTA‐free, 100× in DMSO, HY‐K0010 100×; MedChem Express)] and incubating the samples for 30 min on ice with occasional vortexing. Next, lysates were complemented with 5 mm MgCl_2_ and 100 U Benzonase (Pierce™ Universal Nuclease for Cell Lysis, 88700; Thermo Scientific) and incubated further for 10 min at RT, meanwhile the DNA was sheared using a 26‐gauge (26G) needle. Lysates were clarified by centrifuging them for 10 min at 17 000 ***g*** and 4 °C. The supernatant was diluted twofold with PBS containing PARP‐, PARG‐, and protease inhibitors, and 15 μg biotinylated AbD43647 anti‐MAR antibody (kindly provided by Ivan Matić) was added to allow immunocomplex formation for 3 h on a rotator at 4 °C. Then, 50 μL of Dynabeads™ Streptavidin Magnetic Beads (M‐280; Invitrogen) per sample were prewashed three times in 1 mL of inhibitor‐supplemented PBS buffer and added to the lysates containing the immunocomplexes and incubated on an end‐to‐end rotator for 30 min at RT. The beads were washed four times in 500 μL of inhibitor‐supplemented PBS and MARylated proteins were eluted by adding 100 μL 4% SDS buffer (4% SDS, 50 mm Tris–HCl, pH 7.4, 100 mm NaCl, and 4 mm MgCl_2_) and heating at 95 °C for 5 min. Input lysates and eluates were subjected to Bis‐Tris SDS/PAGE‐based immunoblotting.

### Immunofluorescence

U2OS wild‐type cells were seeded onto 8‐well chambered coverglass and maintained for 48 h under standard culture conditions. Hyperosmotic stress was induced by incubation in 0.4 m D‐sorbitol for 10, 30, or 60 min, followed by recovery in normosmotic medium. For positive control, 10 μm camptothecin was used for 60 min.

Cells were fixed in pre‐chilled methanol/acetone solution (7 : 3) for 15 min at −20 °C and then washed with PBS. Permeabilization was performed with 0.5% Triton X‐100 for 10 min, and then, samples were blocked in 5% FBS for 1 h at room temperature. Samples were incubated overnight at 4 °C with mouse anti‐γH2AX Ser139 (1 : 2000) and subsequently with Alexa Fluor 555‐conjugated anti‐mouse secondary antibody (1 : 700) for 1 h at room temperature (Table 1). Nuclei were counterstained with DAPI (1 μg·mL^−1^) for 10 min.

Z‐stack images were acquired on a spinning‐disk confocal microscope using DAPI and Alexa 555 filter sets and a 40× objective. Image stacks were processed as maximum‐intensity projections with the imagej software.

## Results

### Hyperosmotic stress triggers rapid, stable mono(ADP‐ribosyl)ation and transient poly(ADP‐ribosyl)ation

To determine whether *de novo* ADP‐ribosylation occurs as part of the cellular response to hyperosmotic stress, the lysates of U2OS cells treated with sorbitol for the indicated times were analyzed by western blot using a MAR‐specific antibody (AbD43647) [48] and one detecting both poly‐ and mono‐ADP‐ribose (CST, no.: 89190). A distinct mono(ADP‐ribose) signal appeared at the molecular weight corresponding to the molecular weight of PARP1 within 10 min of sorbitol treatment and remained detectable throughout the duration of hyperosmotic stress (Fig. 1A). In the absence of PARG inhibition, sorbitol‐induced PARylation was barely detectable; however, upon PARGi treatment the PAR signal became apparent, accumulating during hyperosmotic stress and decreased after restoration of normosmotic conditions (Fig. 1B). Together, these data indicate that hyperosmotic stress elicits stable MARylation, whereas PARylation is transient and normally masked by rapid PARG‐dependent turnover.

**Fig. 1:**
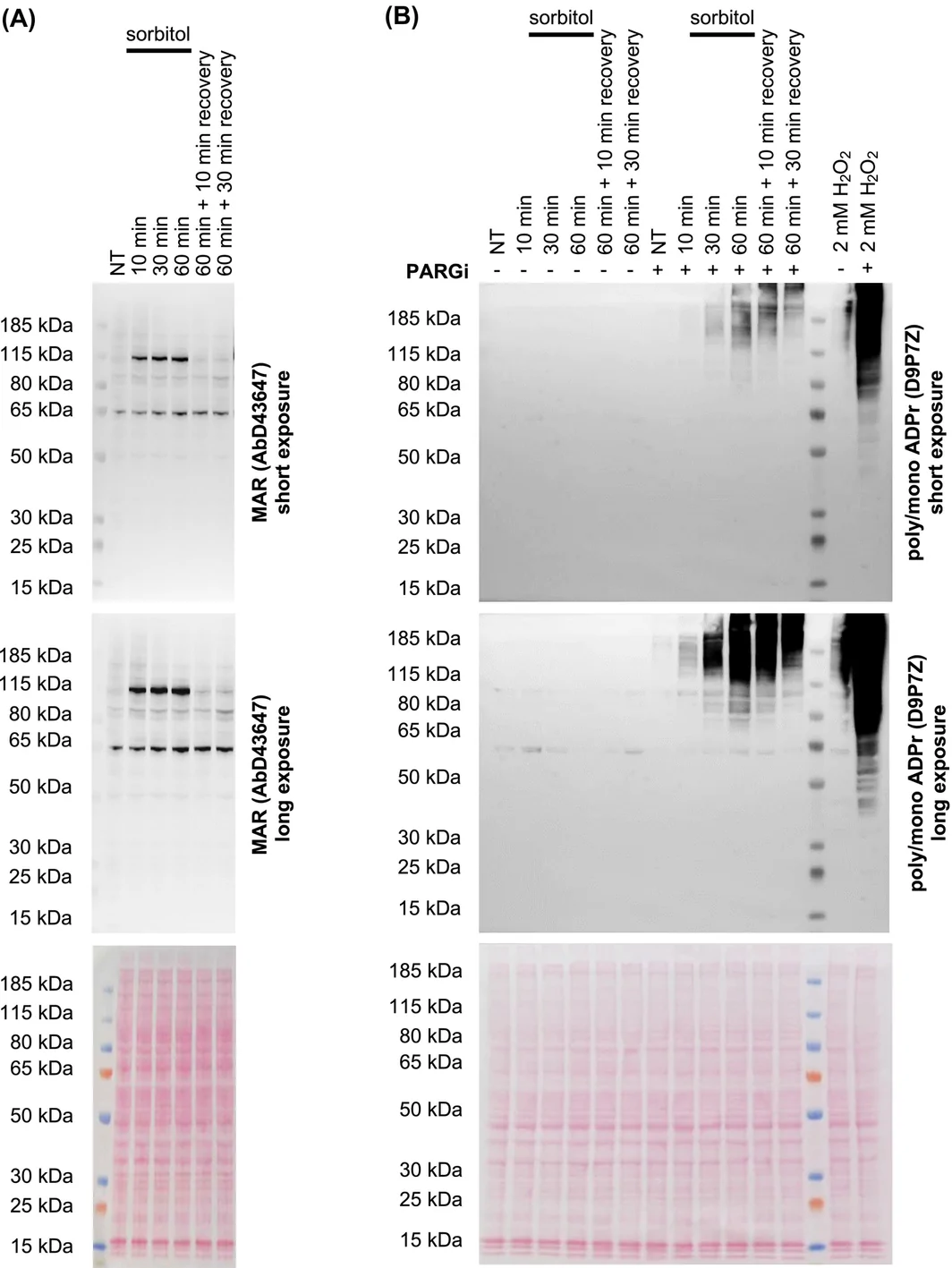
Time dependency of ADP‐ribosylation upon hyperosmotic stress. (A) U2OS wild‐type cells were subjected to 0.4 m D‐sorbitol treatment for 10, 30, or 60 min followed by recovery under normosmotic conditions. In total cell lysate, mono(ADP‐ribosyl)ation was visualized by HRP‐coupled AbD43647. (B) U2OS wild‐type cells were pretreated with PARG inhibitor for 3 h, and then, the cells underwent 0.4 m D‐sorbitol treatment for 10, 30, or 60 min followed by recovery in normosmotic medium. Thereafter, poly(ADP‐ribosyl)ation was detected by the poly/mono‐ADP‐ribose antibody (D9P7Z) from Cell Signalling Technology. U2OS wild‐type cells treated with H_2_O_2_ for 10 min in the presence or absence of PARGi served as a positive control of poly(ADP‐ribosyl)ation. (A, B) Ponceau S staining served as a loading control. Representative western blots of at least three independent experiments. [Correction added on 06 April 2026 after first online publication: The labels “10 min” and “30 min” in Figure 1A were inadvertently transposed and have been corrected in this version.]

Importantly, the MAR signal was rapidly resolved upon restoration of normosmotic conditions, demonstrating that this modification is dynamically controlled. Together, these data establish mono(ADP‐ribosyl)ation as an early and reversible biochemical response to hyperosmotic stress.

### Osmotic stress induces autoMARylation of PARP1

Next, we sought to identify the enzyme responsible for this modification. Given the established roles of PARP1 and PARP2 in chromatin regulation and stress responses [15, 25, 49, 50, 51], we examined their respective contributions to osmotic stress–induced MARylation.

MARylation was measured in sorbitol‐treated U2OS wild‐type, PARP1 knockout, and PARP2 knockout cells on neutral pH gradient gels. While a robust MAR signal was detected in wild‐type and PARP2 knockout cells, it was absent in PARP1 knockout cells (Fig. 2A), indicating that PARP1 is required for this response.

**Fig. 2:**
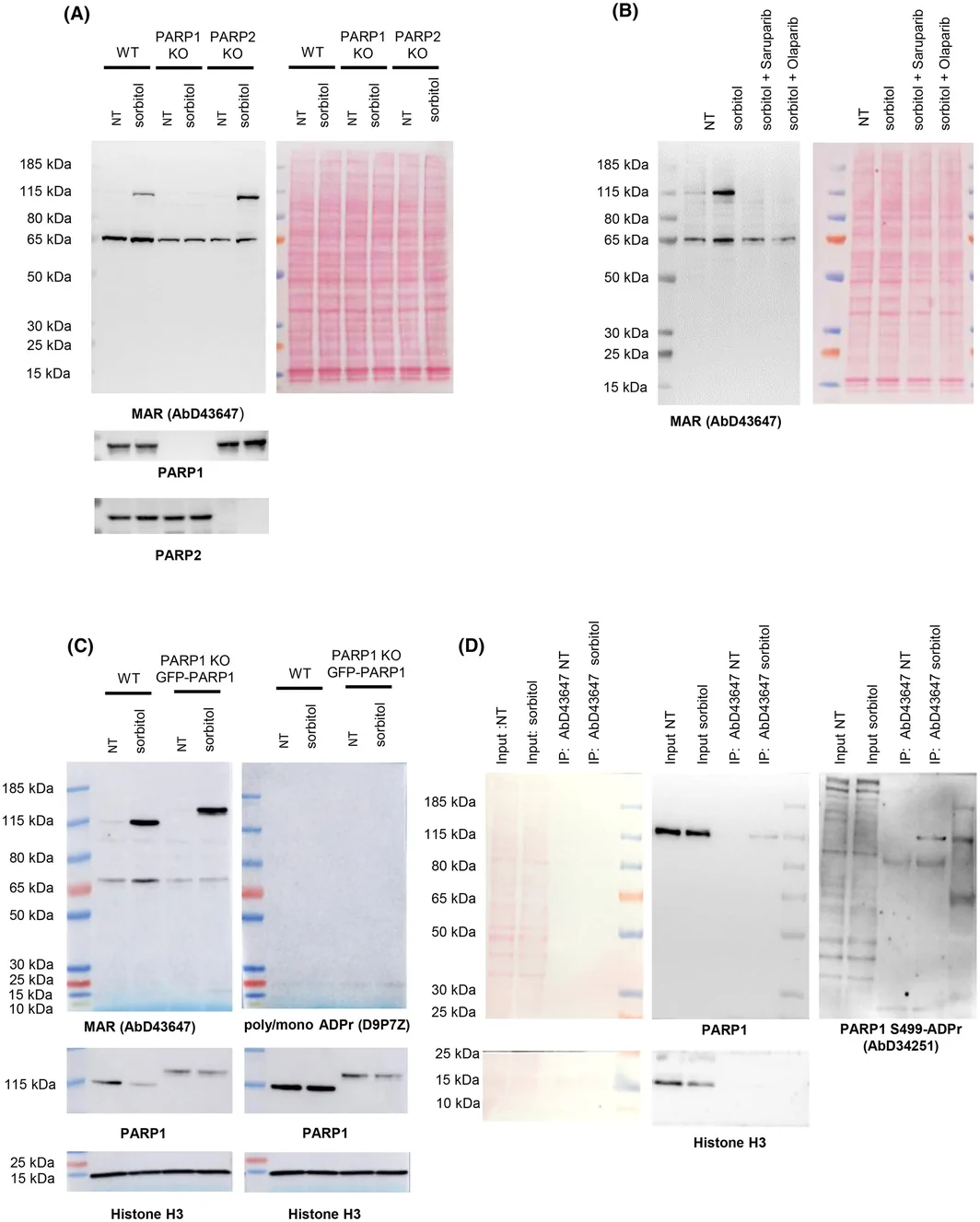
MARylation of PARP1 during hyperosmotic stress. (A) U2OS wild‐type, PARP1 KO, and PARP2 KO cells were exposed to 30‐min‐long D‐sorbitol treatment followed by the collection of total cell lysates and detection of MAR by HRP‐coupled AbD43647. PARP1 and PARP2 western blot verified the knockout status of the used cell lines. (B) U2OS wild‐type cells were pretreated with 5 μm Olaparib, 5 nm Saruparib, or DMSO, followed by exposure to 0.4 m D‐sorbitol for 30 min together with inhibitors. MARylation was detected by HRP‐coupled AbD43647. (C) U2OS wild‐type and GFP‐PARP1 expressing PARP1 KO cells were treated with D‐sorbitol for 30 min. Cell lysates were probed with HRP‐coupled AbD43647 and the poly/mono‐ADP‐ribose antibody (CSL, D9P7Z). (D) Streptavidin pulldown of biotinylated AbD43647 immunoprecipitates from untreated or D‐sorbitol‐treated wild‐type U2OS cells was performed. Input lysates and SDS eluates (IP) from streptavidin magnetic beads were analyzed by immunoblotting with anti‐PARP1 and anti‐PARP1 S499‐MAR (AbD35251)–HRP antibodies. (A, B, D) Ponceau S staining served as a loading control. Representative western blots of at least three independent experiments.

To determine whether PARP1 catalytic activity was necessary, wild‐type cells were pretreated with the PARP inhibitors Olaparib or Saruparib prior to and during sorbitol exposure. Olaparib is a clinically approved PARP1/2 inhibitor, whereas Saruparib is a highly selective PARP1 inhibitor that preferentially inhibits PARP1 catalytic activity at low concentrations. Both inhibitors abolished osmotic stress–induced MARylation (Fig. 2B), confirming that PARP1 is the enzyme mediating MARylation during hyperosmotic stress.

To address whether the MARylated protein corresponds to PARP1 automodification in response to osmotic stress, we applied two approaches. First, we demonstrated that triggering osmotic stress in U2OS PARP1 KO cells expressing mEGFP‐PARP1 resulted in MARylation but no PARylation corresponding to EGFP‐PARP1 with higher molecular weight while the approximately 110 kDa band corresponding to endogenous PARP1 was missing (Fig. 2C). In addition, we performed immunoprecipitation followed by immunoblotting. MARylated proteins were enriched using a biotinylated anti–mono‐ADP‐ribose antibody (AbD43647) and then probed by western blot with either anti‐PARP1 or an antibody recognizing mono‐ADP‐ribosylated PARP1 at S499 (anti–PARP1 S499‐ADPr; AbD34251) [48]. Both PARP1 and mono‐ADP‐ribosylated PARP1 at S499 were detected only in MAR pulldowns prepared from sorbitol‐treated lysates, consistent with stress–induced PARP1 MARylation (Fig. 2D). These data support that autoMARylation of PARP1 represents an early response to osmotic stress.

### Osmotic stress induces HPF1‐dependent, O‐glycosidic MARylation

We next investigated the chemical nature of the osmotic stress–induced MARylation and the involvement of the PARP1 co‐factor HPF1 [44]. Heat and hydroxylamine (HA) treatments were used to distinguish chemically labile acyl‐ester ADP‐ribose linkages on glutamate/aspartate residues from the chemically stable O‐glycosidic linkages generated by PARP1 in the presence of the HPF1 co‐factor [33, 49, 52].

Cell lysates from sorbitol‐treated cells were subjected to heating at 95 °C for 5 min or incubation with 1 m HA for 2 h at room temperature. In parallel, lysates from H_2_O_2_‐treated wild‐type and HPF1 knockout cells were used as controls. As expected, hydroxylamine treatment removed glutamate/aspartate‐linked MARylation in HPF1‐deficient cells but had no effect on serine‐linked MARylation in wild‐type cells (Fig. 3A). Notably, in wild‐type cells, osmotic stress–induced MARylation was resistant to both heat and HA treatment, indicating the presence of O‐glycosidic ADP‐ribose linkages. Consistently, no MARylation was observed in HPF1 knockout cells following sorbitol treatment, demonstrating that HPF1 is required for osmotic stress–induced MARylation.

**Fig. 3:**
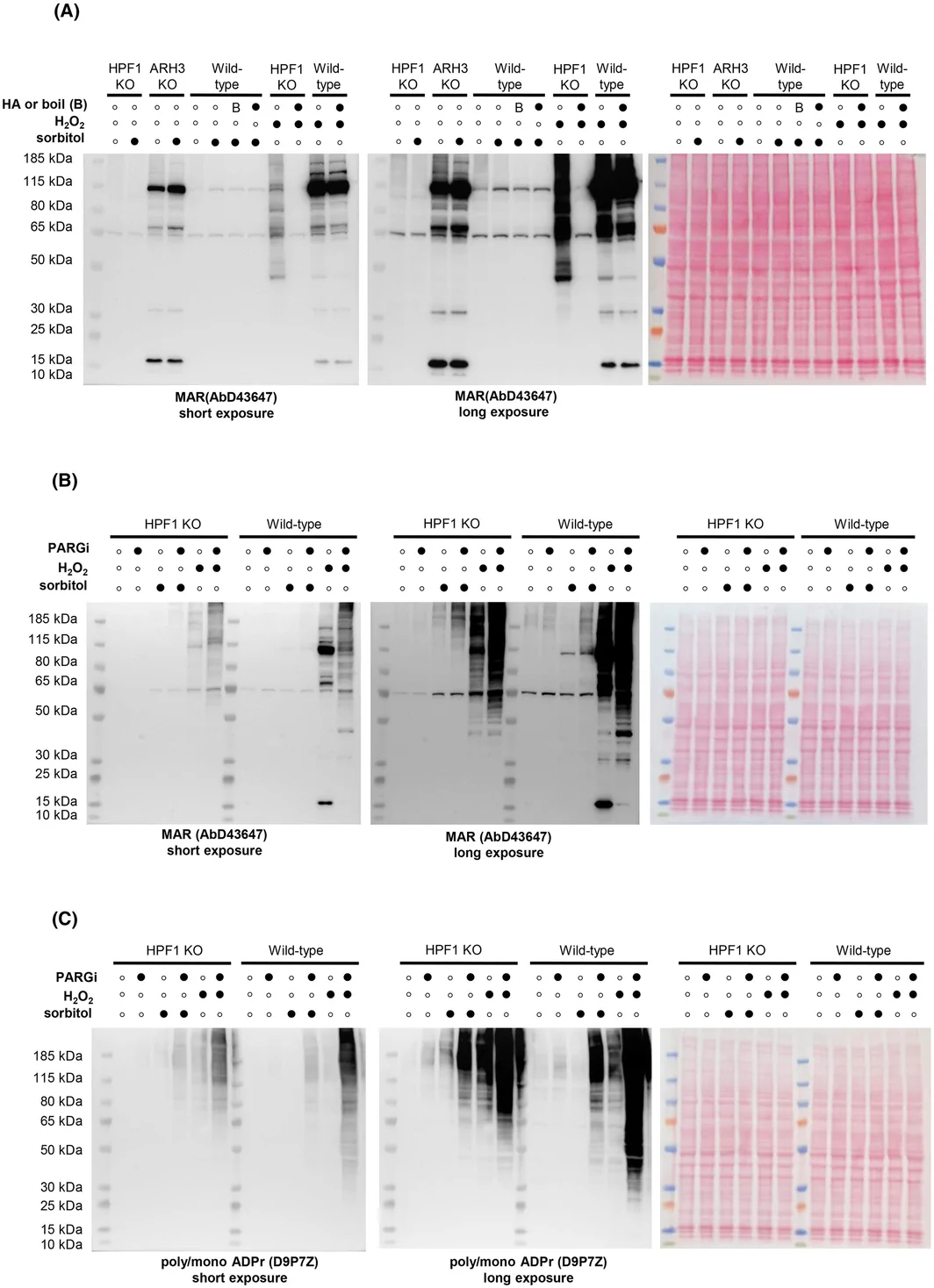
Hyperosmotic stress–induced HPF1‐dependent MARylation and HPF1‐independent PARylation. (A) U2OS wild‐type, HPF1 KO and ARH3 KO cells were subjected to 30 min 0.4 m D‐sorbitol treatment. Total cell lysate of sorbitol‐treated wild‐type U2OS was incubated in the presence of 1 m hydroxylamine (HA) for 2 h at RT, or it was heated at 95 °C for 5 min. As controls of HA treatment, U2OS wild‐type and HPF1 KO cells were exposed to 2 mm H_2_O_2_‐induced oxidative stress followed by incubation with 1 m HA for 2 h at RT. MARylation was detected in western blot using HRP‐coupled Abd43647. (B) U2OS wild‐type or HPF1 KO cells were pretreated with 10 μm PARG inhibitor for 3 h or left untreated, followed by 30 min 0.4 m D‐sorbitol or 10 min 2 mm H_2_O_2_ treatment in the presence or absence of PARG inhibitor. MARylation was visualized by HRP‐coupled Abd43647 (C) U2OS wild‐type or HPF1 KO cells were pretreated with 10 μm PARG inhibitor for 3 h or left untreated, followed by 30 min 0.4 m D‐sorbitol or 10 min 2 mm H_2_O_2_ treatment in the presence or absence of PARG inhibitor. Thereafter, poly(ADP‐ribosyl)ation was detected by the poly/mono‐ADP‐ribose antibody (D9P7Z) from Cell Signalling Technology. (A–C) Ponceau S staining was used as a loading control. Representative western blot of at least three independent experiments.

To further assess the regulation of this modification, we examined ARH3 knockout cells, which are deficient in removing O‐glycosidic ADP‐ribose. ARH3‐deficient cells exhibited elevated basal MAR levels, and sorbitol treatment resulted in only a modest further increase, consistent with reduced ADP‐ribose turnover capacity and partial saturation of PARP1 modification sites.

Given that PARG has been shown to remove the terminal ADP‐ribose from glutamate, aspartate, and tyrosine [17, 33, 34], we addressed whether inhibiting PARG could reveal PARP1 MARylation in HPF1 KO cells. Upon H_2_O_2_ treatment, used as a positive control, HPF1 KO cells displayed MARylation, which became more pronounced in the presence of PARGi (Fig. 3B). In contrast, sorbitol failed to induce detectable MARylation in HPF1 KO cells even with PARGi (Fig. 3B), indicating that osmotic stress–induced PARP1 MARylation is strictly HPF1 dependent and not unmasked by blocking PARG.

We have established that, although masked by PARG activity, osmotic stress also elicits PARylation. One possible explanation for the prominent MAR signal is rapid trimming of stress–induced PAR back to MAR by PARG. In accordance with this concept, in H_2_O_2_‐treated wild‐type cells, both histone and PARP1 MARylation diminished in the presence of PARGi, in line with MARylated sites being further extended into PAR when ADP‐ribose turnover is blocked (Fig. 3B). Strikingly, however, sorbitol‐induced PARP1 autoMARylation did not decrease upon PARG inhibition and remained essentially unchanged (Fig. 3B). This suggests that osmotic stress primarily induces mono(ADP‐ribosyl)ation on PARP1 that is not efficiently converted into PAR, even when PARG activity is inhibited.

In parallel, we examined osmotic stress–induced PARylation in HPF1 KO cells in the presence of PARGi. In HPF1 KO cells, both sorbitol and H_2_O_2_ elicited higher PARylation than in wild‐type—consistent with HPF1 shifting PARP1 from chain elongation toward serine‐linked ADP‐ribosylation [16, 43, 53]—and PARG inhibition further increased PAR levels under both conditions (Fig. 3C).

Together, these results establish that hyperosmotic stress induces a tightly regulated, HPF1‐dependent mono(ADP‐ribosyl)ation of PARP1 with O‐glycosidic linkage chemistry.

### Short‐term hyperosmotic stress perturbs replication dynamics without inducing DNA damage

Given that PARP1–HPF1‐mediated ADP‐ribosylation is closely linked to genome surveillance and replication‐associated processes [25, 48, 49, 50, 51], we next assessed whether hyperosmotic stress under these conditions impacts cell survival or activates markers of DNA damage and replication stress.

To examine potential DNA damage or replication stress, we analyzed γH2AX levels by immunofluorescence microscopy and CHK1 phosphorylation at Ser317 by western blot. Phosphorylation of histone H2AX (γH2AX) marks the presence of DNA double‐strand breaks, while phosphorylation of CHK1 at Ser317 is a hallmark of ATR‐mediated checkpoint activation in response to replication stress [54, 55]. We did not observe the formation of γH2AX signal in response to hyperosmotic stress suggesting the absence of significant DNA double‐strand breaks (Fig. 4A). In contrast, phosphorylated CHK1 was not detected during hyperosmotic stress but became evident during the recovery phase following the restoration of normosmotic conditions, indicating transient perturbation of replication (Fig. 4B).

**Fig. 4:**
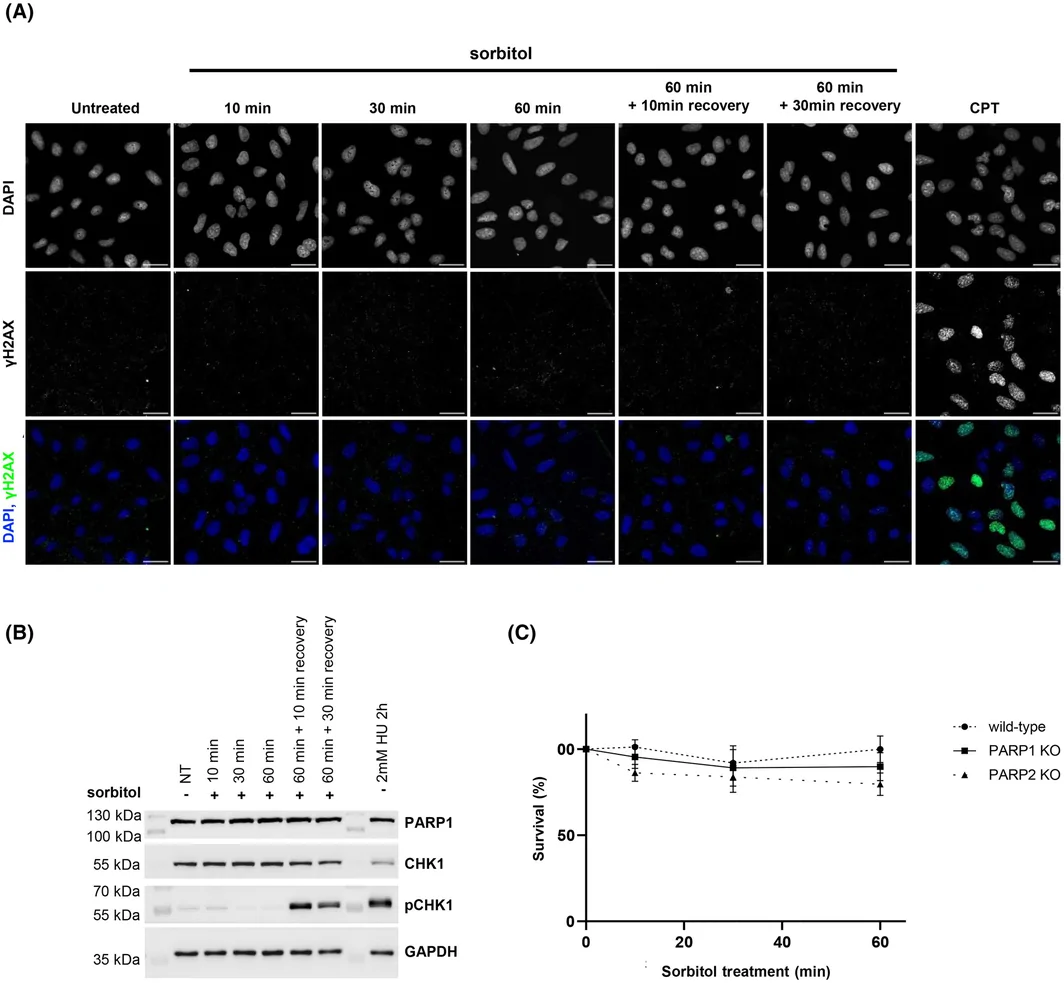
Effect of hyperosmotic stress on cell survival and CHK1 activation. (A) Immunofluorescent staining of phosphorylated histone H2AX (yH2AX) in U2OS wild‐type cells exposed to 10, 30, or 60 min hyperosmotic stress in 0.4 m D‐sorbitol followed by recovery in normosmotic medium. Cells were treated with 10 μm camptothecin (CPT) for 1 h for positive control. Representative images of at least three independent experiments. Scale bar: 35 μm. (B) Representative western blot of at least three independent experiments to detect pCHK1(S317), CHK1, and PARP1 in U2OS wild‐type cells treated with 0.4 m D‐sorbitol for 10, 30, or 60 min followed by recovery in normosmotic media. GAPDH was used as a loading control. (C) Time course of cell survival in U2OS wild‐type, PARP1 KO and PARP2 KO cells during 0.4 m D‐sorbitol treatment. Wild‐type and knockout U2OS cells were treated with 0.4 m D‐sorbitol for the indicated time and subsequently cultured for 7 days before measuring the fluorescence of resorufin. Error bars are SD (*n* = 3).

To determine whether the used osmotic stress compromised cell survival, U2OS wild‐type, PARP1 knockout, and PARP2 knockout cells were treated with 0.4 m D‐sorbitol for up to 60 min, and cell viability was evaluated using a resazurin‐based assay. No significant reduction in viability was observed in any of the tested cell lines (Fig. 4C), indicating that short‐term hyperosmotic stress is well tolerated and does not induce acute cytotoxicity.

These results suggest that PARP1–HPF1‐mediated MARylation represents an early adaptive response to hyperosmotic stress rather than a consequence of genotoxic damage or cytotoxic stress.

## Discussion

In this study, we identify hyperosmotic stress as a trigger of rapid and reversible PARP1‐dependent mono(ADP‐ribosyl)ation. Although PARP1 is capable of polymer synthesis, sorbitol‐induced PAR is barely detectable under basal conditions but becomes apparent upon PARG inhibition, indicating that PAR is formed yet rapidly turned over by PARG. Notably, PARP1 autoMARylation is lost in HPF1‐knockout cells, establishing that the response requires HPF1. The hyperosmotic stress–induced MARylation is resistant to hydroxylamine and heat treatment, consistent with an O‐glycosidic linkage, together defining an HPF1‐dependent PARP1 mono(ADP‐ribosyl)ation response to hyperosmotic stress.

By adding D‐sorbitol, a nonionic and largely membrane‐impermeant osmolyte, we increased extracellular osmolality without increasing extracellular salt, thereby inducing rapid cell shrinkage and macromolecular crowding while minimizing salt‐specific ionic stress. In line with previous reports, sorbitol‐induced hyperosmosis did not result in detectable γH2AX accumulation [56] supporting the conclusion that the applied conditions do not induce overt DNA double‐strand breaks. Notably, the well‐studied sodium‐chloride‐induced osmotic stress leads to γH2AX foci formation; however, it has additional ionic toxicity, adding several layers of complexity beyond increased osmolality [57].

High NaCl stress has been shown to cause MRE11 export from the nucleus thereby suppressing ATR‐CHK1 activation during the stress itself. When osmolality returns to normal, the checkpoint becomes responsive again and ATR‐CHK1 activation can occur [58]. In contrast, sorbitol‐induced hyperosmosis affects the ATR‐CHK1 axis in a highly cell‐type‐dependent manner [13, 56]. Notably, our observation that CHK1 phosphorylation is restricted to the recovery phase is consistent with a previous report showing that MEF cells display elevated pCHK1 only during recovery from severe hyperosmotic stress, but not during the high‐dose sorbitol treatment itself [59]. Altogether, these observations indicate that PARP1 activation during hyperosmosis occurs independently of classical genotoxic stress.

A possible explanation for PARP1‐driven stable MARylation can be linked to structural changes of chromatin upon osmotic stress. Disruption of chromatin organization and the accumulation of positively supercoiled DNA were described in *Saccharomyces cerevisiae* as a result of topological stress occurring within 30 s after adding sorbitol [60]. Overwound DNA can be unwound and relaxed, and secondary redistribution of the torsional strain can lead to negative supercoiling, which promotes the formation of structures such as G4 DNA. PARP1 was shown to bind G4 structure *in vitro* [61] and to be activated by cruciform structures, which are associated with negative torsional stress [62]. Based on these data, one can speculate that PARP1 may bind and is activated by non‐B DNA structures linked to hyperosmosis‐induced mechanical stress.

The physiological relevance of hyperosmotic stress is also reflected at the organismal level. In a recent whole‐genome analysis of hairy‐footed jerboas inhabiting hyper‐arid deserts, *HPF1* was among the genes showing signatures of positive selection based on shifts in SNP frequencies between hyper‐arid and semi‐desert populations [63]. Although the study did not functionally characterize these variants, *HPF1* was grouped with genes associated with responses to multiple environmental stressors, including severe dehydration and osmotic imbalance [63]. Our findings complement these observations by providing a biochemical and cell‐biological description of HPF1‐dependent MARylation during osmotic stress, thereby suggesting a potential mechanistic basis through which HPF1 activity may contribute to osmostress adaptation.

In summary, our data establish hyperosmotic stress as a nongenotoxic trigger of rapid and reversible PARP1 activation that is also dependent on HPF1 to generate a prominent mono(ADP‐ribose) signature. This expands the repertoire of physiological inputs that engage the PARP1–HPF1 axis beyond canonical DNA damage signaling and suggests that ADP‐ribosylation may participate in chromatin adaptation to acute mechanical/osmotic challenge. Key questions for future work will be to define the initiating molecular cues that activate PARP1 during hyperosmotic stress, map the relevant modification sites on PARP1 and other targets, and determine how this pathway influences chromatin function and recovery programs after osmotic stress.

## Author contributions

AGK, MM, AC, and GT contributed to the study conception and design and manuscript preparation. VI contributed to the resources. AGK, MM, RV, LH, RP, AK, and AC contributed to the data collection. AGK, LH, SH, NDL, IA, AC, GT contributed to the analysis and interpretation of results. All authors reviewed the results and approved the final version of the manuscript.

## Data Availability

The data supporting the results of this study are available from the corresponding authors upon reasonable request.

## References

[1] Brocker C , Thompson DC and Vasiliou V (2012) The role of hyperosmotic stress in inflammation and disease. Biomol Concepts 3, 345–364. 10.1515/bmc-2012-0001 PMC3438915 22977648

[2] Saito H and Posas F (2012) Response to hyperosmotic stress. Genetics 192, 289–318. 10.1534/genetics.112.140863 PMC3454867 23028184

[3] Woo SK and Kwon HM (2002) Adaptation of kidney medulla to hypertonicity: role of the transcription factor TonEBP. Int Rev Cytol 215, 189–202. 10.1016/s0074-7696(02)15009-1 11952228

[4] Probst S , Romanova N , Herbrechter R , Kern T , Bergmeier M , Lee W‐K and Thévenod F (2025) Hyperosmolarity‐induced activation of PIEZO1 engages detrimental calcium/oxidative stress signaling and adaptive catalase response in renal inner medullary collecting duct (mIMCD3) cells. Biochim Biophys Acta 1872, 120041. 10.1016/j.bbamcr.2025.120041 40780677

[5] Harrell CR , Feulner L , Djonov V , Pavlovic D and Volarevic V (2023) The molecular mechanisms responsible for tear hyperosmolarity‐induced pathological changes in the eyes of dry eye disease patients. Cells 12, 2755. 10.3390/cells12232755 PMC10706334 38067183

[6] Haussinger D (1993) Cellular hydration state: an important determinant of protein catabolism in health and disease. Lancet 341, 1330–1332. 10.1016/0140-6736(93)90828-5 8098459

[7] Wang H , He Z , Li J , Lin C , Li H , Jin P and Chen C (2021) Early plasma osmolality levels and clinical outcomes in children admitted to the pediatric intensive care unit: a single‐center cohort study. Front Pediatr 9, 745204. 10.3389/fped.2021.745204 PMC8481873 34604144

[8] Shapiro L and Dinarello CA (1995) Osmotic regulation of cytokine synthesis in vitro. Proc Natl Acad Sci USA 92, 12230–12234. 10.1073/pnas.92.26.12230 PMC40330 8618875

[9] Sumida TS (2023) Hyperosmotic stress response regulates interstitial homeostasis and pathogenic inflammation. J Biochem 173, 159–166. 10.1093/jb/mvad009 36722164

[10] Vertii A (2021) Stress as a chromatin landscape architect. Front Cell Dev Biol 9, 790138. 10.3389/fcell.2021.790138 PMC8712864 34970548

[11] Burg MB , Ferraris JD and Dmitrieva NI (2007) Cellular response to hyperosmotic stresses. Physiol Rev 87, 1441–1474. 10.1152/physrev.00056.2006 17928589

[12] Miermont A , Waharte F , Hu S , McClean MN , Bottani S , Léon S and Hersen P (2013) Severe osmotic compression triggers a slowdown of intracellular signaling, which can be explained by molecular crowding. Proc Natl Acad Sci USA 110, 5725–5730. 10.1073/pnas.1215367110 PMC3619343 23493557

[13] Canat A , Atilla D and Torres‐Padilla M (2023) Hyperosmotic stress induces 2‐cell‐like cells through ros and atr signaling. EMBO Rep 24, e56194. 10.15252/embr.202256194 PMC10481651 37432066

[14] Suskiewicz MJ , Prokhorova E , Rack JGM and Ahel I (2023) ADP‐ribosylation from molecular mechanisms to therapeutic implications. Cell 186, 4475–4495. 10.1016/j.cell.2023.08.030 PMC10789625 37832523

[15] Bonfiglio JJ , Fontana P , Zhang Q , Colby T , Gibbs‐Seymour I , Atanassov I , Bartlett E , Zaja R , Ahel I and Matic I (2017) Serine ADP‐ribosylation depends on HPF1. Mol Cell 65, 932–940.e6. 10.1016/j.molcel.2017.01.003 PMC5344681 28190768

[16] Suskiewicz MJ , Zobel F , Ogden TEH , Fontana P , Ariza A , Yang J‐C , Zhu K , Bracken L , Hawthorne WJ , Ahel D *et al*. (2020) HPF1 completes the PARP active site for DNA damage‐induced ADP‐ribosylation. Nature 579, 598–602. 10.1038/s41586-020-2013-6 PMC7104379 32028527

[17] Rack JGM , Voorneveld J , Longarini EJ , Wijngaarden S , Zhu K , Peters A , Sia JJ , Prokhorova E , Ahel D , Matić I *et al*. (2024) Reversal of tyrosine‐linked ADP‐ribosylation by ARH3 and PARG. J Biol Chem 300, 107838. 10.1016/j.jbc.2024.107838 PMC11541775 39342999

[18] Teloni F and Altmeyer M (2016) Readers of poly(ADP‐ribose): designed to be fit for purpose. Nucleic Acids Res 44, 993–1006. 10.1093/nar/gkv1383 PMC4756826 26673700

[19] Alemasova EE and Lavrik OI (2019) Poly(ADP‐ribosyl)ation by PARP1: reaction mechanism and regulatory proteins. Nucleic Acids Res 47, 3811–3827. 10.1093/nar/gkz120 PMC6486540 30799503

[20] Eisemann T and Pascal JM (2020) Poly(ADP‐ribose) polymerase enzymes and the maintenance of genome integrity. Cell Mol Life Sci 77, 19–33. 10.1007/s00018-019-03366-0 PMC11104942 31754726

[21] Aberle L , Krüger A , Reber JM , Lippmann M , Hufnagel M , Schmalz M , Trussina IREA , Schlesiger S , Zubel T , Schütz K *et al*. (2020) PARP1 catalytic variants reveal branching and chain length‐specific functions of poly(ADP‐ribose) in cellular physiology and stress response. Nucleic Acids Res 48, 10015–10033. 10.1093/nar/gkaa590 PMC7544232 32667640

[22] Pascal JM and Ellenberger T (2015) The rise and fall of poly(ADP‐ribose): an enzymatic perspective. DNA Repair 32, 10–16. 10.1016/j.dnarep.2015.04.008 PMC4522361 25963443

[23] Caldecott KW (2014) Protein ADP‐ribosylation and the cellular response to DNA strand breaks. DNA Repair 19, 108–113. 10.1016/j.dnarep.2014.03.021 24755000

[24] Duma L and Ahel I (2023) The function and regulation of ADP‐ribosylation in the DNA damage response. Biochem Soc Trans 51, 995–1008. 10.1042/BST20220749 PMC10317172 37171085

[25] Rudolph J and Luger K (2025) Histone PARylation factor 1: a review of its role in the DNA damage response. Nucleic Acids Res 53, gkaf1170. 10.1093/nar/gkaf1170 PMC12596734 41206044

[26] Zong W , Gong Y , Sun W , Li T and Wang Z‐Q (2022) PARP1: liaison of chromatin remodeling and transcription. Cancer 14, 4162. 10.3390/cancers14174162 PMC9454564 36077699

[27] Longarini EJ , Dauben H , Locatelli C , Wondisford AR , Smith R , Muench C , Kolvenbach A , Lynskey ML , Pope A , Bonfiglio JJ *et al*. (2023) Modular antibodies reveal DNA damage‐induced mono‐ADP‐ribosylation as a second wave of PARP1 signaling. Mol Cell 83, 1743–1760.e11. 10.1016/j.molcel.2023.03.027 PMC10205078 37116497

[28] Kloet MS , Chatrin C , Mukhopadhyay R , Van Tol BDM , Smith R , Rotman SA , Tjokrodirijo RTN , Zhu K , Gorelik A , Maginn L *et al*. (2025) Identification of RNF114 as ADPr‐Ub reader through non‐hydrolysable ubiquitinated ADP‐ribose. Nat Commun 16, 6319. 10.1038/s41467-025-61111-7 PMC12241653 40634336

[29] Slade D , Dunstan MS , Barkauskaite E , Weston R , Lafite P , Dixon N , Ahel M , Leys D and Ahel I (2011) The structure and catalytic mechanism of a poly(ADP‐ribose) glycohydrolase. Nature 477, 616–620. 10.1038/nature10404 PMC3184140 21892188

[30] Fontana P , Bonfiglio JJ , Palazzo L , Bartlett E , Matic I and Ahel I (2017) Serine ADP‐ribosylation reversal by the hydrolase ARH3. elife 6, e28533. 10.7554/eLife.28533 PMC5552275 28650317

[31] Sharifi R , Morra R , Denise Appel C , Tallis M , Chioza B , Jankevicius G , Simpson MA , Matic I , Ozkan E , Golia B *et al*. (2013) Deficiency of terminal ADP‐ribose protein glycohydrolase TARG1/C6orf130 in neurodegenerative disease. EMBO J 32, 1225–1237. 10.1038/emboj.2013.51 PMC3642678 23481255

[32] Rosenthal F , Feijs KLH , Frugier E , Bonalli M , Forst AH , Imhof R , Winkler HC , Fischer D , Caflisch A , Hassa PO *et al*. (2013) Macrodomain‐containing proteins are new mono‐ADP‐ribosylhydrolases. Nat Struct Mol Biol 20, 502–507. 10.1038/nsmb.2521 23474714

[33] Longarini EJ and Matić I (2024) Preserving ester‐linked modifications reveals glutamate and aspartate mono‐ADP‐ribosylation by PARP1 and its reversal by PARG. Nat Commun 15, 4239. 10.1038/s41467-024-48314-0 PMC11102441 38762517

[34] Tashiro K , Wijngaarden S , Mohapatra J , Rack JGM , Ahel I , Filippov DV and Liszczak G (2023) Chemoenzymatic and synthetic approaches to investigate aspartate‐ and glutamate‐ADP‐ribosylation. J Am Chem Soc 145, 14000–14009. 10.1021/jacs.3c03771 PMC11065122 37315125

[35] Talhaoui I , Lebedeva NA , Zarkovic G , Saint‐Pierre C , Kutuzov MM , Sukhanova MV , Matkarimov BT , Gasparutto D , Saparbaev MK , Lavrik OI *et al*. (2016) Poly(ADP‐ribose) polymerases covalently modify strand break termini in DNA fragments *in vitro* . Nucleic Acids Res 44, 9279–9295. 10.1093/nar/gkw675 PMC5100588 27471034

[36] Matta E , Kiribayeva A , Khassenov B , Matkarimov BT and Ishchenko AA (2020) Insight into DNA substrate specificity of PARP1‐catalysed DNA poly(ADP‐ribosyl)ation. Sci Rep 10, 3699. 10.1038/s41598-020-60631-0 PMC7048826 32111879

[37] Wondisford AR , Lee J , Lu R , Schuller M , Groslambert J , Bhargava R , Schamus‐Haynes S , Cespedes LC , Opresko PL , Pickett HA *et al*. (2024) Deregulated DNA ADP‐ribosylation impairs telomere replication. Nat Struct Mol Biol 31, 791–800. 10.1038/s41594-024-01279-6 PMC11102865 38714889

[38] Singatulina AS , Sukhanova MV , Desforges B , Joshi V , Pastré D and Lavrik OI (2022) PARP1 activation controls stress granule assembly after oxidative stress and DNA damage. Cells 11, 3932. 10.3390/cells11233932 PMC9740212 36497190

[39] Catara G , Grimaldi G , Schembri L , Spano D , Turacchio G , Lo Monte M , Beccari AR , Valente C and Corda D (2017) PARP1‐produced poly‐ADP‐ribose causes the PARP12 translocation to stress granules and impairment of Golgi complex functions. Sci Rep 7, 14035. 10.1038/s41598-017-14156-8 PMC5656619 29070863

[40] Isabelle M , Gagné J‐P , Gallouzi I‐E and Poirier GG (2012) Quantitative proteomics and dynamic imaging reveal that G3BP‐mediated stress granule assembly is poly(ADP‐ribose)‐dependent following exposure to MNNG‐induced DNA alkylation. J Cell Sci 4555–4566. 10.1242/jcs.106963 22767504

[41] Watanabe K , Morishita K , Zhou X , Shiizaki S , Uchiyama Y , Koike M , Naguro I and Ichijo H (2021) Cells recognize osmotic stress through liquid–liquid phase separation lubricated with poly(ADP‐ribose). Nat Commun 12, 1353. 10.1038/s41467-021-21614-5 PMC7921423 33649309

[42] Watanabe K , Umeda T , Niwa K , Naguro I and Ichijo H (2018) A PP6‐ASK3 module coordinates the bidirectional cell volume regulation under osmotic stress. Cell Rep 22, 2809–2817. 10.1016/j.celrep.2018.02.045 29539411

[43] Prokhorova E , Agnew T , Wondisford AR , Tellier M , Kaminski N , Beijer D , Holder J , Groslambert J , Suskiewicz MJ , Zhu K *et al*. (2021) Unrestrained poly‐ADP‐ribosylation provides insights into chromatin regulation and human disease. Mol Cell 81, 2640–2655.e8. 10.1016/j.molcel.2021.04.028 PMC8221567 34019811

[44] Gibbs‐Seymour I , Fontana P , Rack JGM and Ahel I (2016) HPF1/C4orf27 is a PARP‐1‐interacting protein that regulates PARP‐1 ADP‐ribosylation activity. Mol Cell 62, 432–442. 10.1016/j.molcel.2016.03.008 PMC4858568 27067600

[45] Ronson GE , Piberger AL , Higgs MR , Olsen AL , Stewart GS , McHugh PJ , Petermann E and Lakin ND (2018) PARP1 and PARP2 stabilise replication forks at base excision repair intermediates through Fbh1‐dependent Rad51 regulation. Nat Commun 9, 746. 10.1038/s41467-018-03159-2 PMC5821833 29467415

[46] Juhász S , Smith R , Schauer T , Spekhardt D , Mamar H , Zentout S , Chapuis C , Huet S and Timinszky G (2020) The chromatin remodeler ALC1 underlies resistance to PARP inhibitor treatment. Sci Adv 6, eabb8626. 10.1126/sciadv.abb8626 PMC11206534 33355125

[47] Dauben H and Matic I (2023) Immunoprecipitation using mono‐ADP‐ribosylation‐specific antibodies. Methods in Molecular Biology 2609, 135–146. 10.1007/978-1-0716-2891-1_9 36515834

[48] Bonfiglio JJ , Leidecker O , Dauben H , Longarini EJ , Colby T , San Segundo‐Acosta P , Perez KA and Matic I (2020) An HPF1/PARP1‐based chemical biology strategy for exploring ADP‐ribosylation. Cell 183, 1086–1102.e23. 10.1016/j.cell.2020.09.055 33186521

[49] Palazzo L , Leidecker O , Prokhorova E , Dauben H , Matic I and Ahel I (2018) Serine is the major residue for ADP‐ribosylation upon DNA damage. elife 7, e34334. 10.7554/eLife.34334 PMC5837557 29480802

[50] Kumamoto S , Nishiyama A , Chiba Y , Miyashita R , Konishi C , Azuma Y and Nakanishi M (2021) HPF1‐dependent PARP activation promotes LIG3‐XRCC1‐mediated backup pathway of Okazaki fragment ligation. Nucleic Acids Res 49, 5003–5016. 10.1093/nar/gkab269 PMC8136790 33872376

[51] Genois M‐M , Gagné J‐P , Yasuhara T , Jackson J , Saxena S , Langelier M‐F , Ahel I , Bedford MT , Pascal JM , Vindigni A *et al*. (2021) CARM1 regulates replication fork speed and stress response by stimulating PARP1. Mol Cell 81, 784–800.e8. 10.1016/j.molcel.2020.12.010 PMC7897296 33412112

[52] Moss J , Yost DA and Stanley SJ (1983) Amino acid‐specific ADP‐ribosylation. J Biol Chem 258, 6466–6470. 6304041

[53] Langelier M‐F , Billur R , Sverzhinsky A , Black BE and Pascal JM (2021) HPF1 dynamically controls the PARP1/2 balance between initiating and elongating ADP‐ribose modifications. Nat Commun 12, 6675. 10.1038/s41467-021-27043-8 PMC8602370 34795260

[54] Zhao H and Piwnica‐Worms H (2001) ATR‐mediated checkpoint pathways regulate phosphorylation and activation of human Chk1. Mol Cell Biol 21, 4129–4139. 10.1128/MCB.21.13.4129-4139.2001 PMC87074 11390642

[55] Wilsker D , Petermann E , Helleday T and Bunz F (2008) Essential function of Chk1 can be uncoupled from DNA damage checkpoint and replication control. Proc Natl Acad Sci USA 105, 20752–20757. 10.1073/pnas.0806917106 PMC2634938 19091954

[56] Kumar A , Mazzanti M , Mistrik M , Kosar M , Beznoussenko GV , Mironov AA , Garrè M , Parazzoli D , Shivashankar GV , Scita G *et al*. (2014) ATR mediates a checkpoint at the nuclear envelope in response to mechanical stress. Cell 158, 633–646. 10.1016/j.cell.2014.05.046 PMC4121522 25083873

[57] Dmitrieva NI and Burg MB (2008) Analysis of DNA breaks, DNA damage response, and apoptosis produced by high NaCl. Am J Physiol Renal Physiol 295, F1678–F1688. 10.1152/ajprenal.90424.2008 PMC2604838 18829739

[58] Dmitrieva NI , Cai Q and Burg MB (2004) Cells adapted to high NaCl have many DNA breaks and impaired DNA repair both in cell culture and *in vivo* . Proc Natl Acad Sci USA 101, 2317–2322. 10.1073/pnas.0308463100 PMC356948 14983007

[59] Jobava R , Mao Y , Guan B‐J , Hu D , Krokowski D , Chen C‐W , Shu XE , Chukwurah E , Wu J , Gao Z *et al*. (2021) Adaptive translational pausing is a hallmark of the cellular response to severe environmental stress. Mol Cell 81, 4191–4208.e8. 10.1016/j.molcel.2021.09.029 PMC8559772 34686314

[60] Gittens WH , Allison RM , Wright EM , Brown GGB and Neale MJ (2024) Osmotic disruption of chromatin induces topoisomerase 2 activity at sites of transcriptional stress. Nat Commun 15, 10606. 10.1038/s41467-024-54567-6 PMC11621772 39639049

[61] Soldatenkov VA , Vetcher AA , Duka T and Ladame S (2008) First evidence of a functional interaction between DNA quadruplexes and poly(ADP‐ribose) Polymerase‐1. ACS Chem Biol 3, 214–219. 10.1021/cb700234f 18338862

[62] Lonskaya I , Potaman VN , Shlyakhtenko LS , Oussatcheva EA , Lyubchenko YL and Soldatenkov VA (2005) Regulation of poly(ADP‐ribose) Polymerase‐1 by DNA structure‐specific binding. J Biol Chem 280, 17076–17083. 10.1074/jbc.M413483200 15737996

[63] Peng X , Cheng J , Li H , Feijó A , Xia L , Ge D , Wen Z and Yang Q (2023) Whole‐genome sequencing reveals adaptations of hairy‐footed jerboas (dipus, Dipodidae) to diverse desert environments. BMC Biol 21, 182. 10.1186/s12915-023-01680-5 PMC10469962 37649052

